# Emergency Dispatches for Suicide Attempts during the COVID-19 Pandemic in Okayama, Japan: An Interrupted Time-series Analysis

**DOI:** 10.31662/jmaj.2024-0009

**Published:** 2024-06-03

**Authors:** Yuka Yamamura, Naomi Matsumoto, Soshi Takao, Takashi Yorifuji

**Affiliations:** 1 Department of Epidemiology, Graduate School of Medicine, Dentistry and Pharmaceutical Sciences, Okayama University, Okayama, Japan

**Keywords:** COVID-19, epidemiology, emergency medical dispatch, suicide attempt

## Introduction

Four years have passed since the COVID-19 pandemic began, which has led to a high number of deaths and heavy public health burden worldwide ^(1)^. COVID-19 has also affected trends in other noncommunicable diseases, such as stroke ^(2)^, alcohol-related liver disease and pancreatitis ^(3)^, and cognitive function and mental health among older adults ^(4)^. Concerns have also emerged regarding the influence of the pandemic on suicide, which could be a specific marker of mental distress.

Although the influence of the pandemic on suicide and suicide attempts seems to be inconsistent among countries, several countries including Japan observed an increase in the number of suicides during the early period of the pandemic ^(5)^. In comparison, the suicide rates in the United States did not increase after the start of the pandemic (i.e., 14.7 deaths per 100,000 persons in 2019; 14.1 deaths per 100,000 persons in 2020), whereas those in Japan rose (i.e., 14.6 deaths per 100,000 persons in 2019; 15.4 deaths per 100,000 persons in 2020) ^(6)^. Indeed, several studies from Japan reported an increase in suicide mortality and suicide attempts during the first year of the pandemic (i.e., 2020) among women and middle-aged people ^(7), (8)^; the reasons for such an increase were considered to be associated with work or family-related stress or economic pressure (e.g., job insecurity and reduced income) ^(9), (10), (11)^. Some studies have further explored the long-term impact of the pandemic (i.e., after 2021, more than 2 years after the first occurrence of COVID-19 in Japan) on suicide mortality and demonstrated that suicide rates increased among women and in younger age groups until 2021 ^(9), (10)^.

Previous studies conducted in Japan have explored the impact of the pandemic on suicide mortality in 2020 and 2021 and on suicide attempts in 2020. However, few studies have evaluated the long-term impact of the pandemic (i.e., after 2021) on suicide attempts ^(12), (13)^. This study investigated the incidence of suicide attempts during the COVID-19 pandemic in comparison with the prepandemic period, considering variations across sex and age groups, utilizing data from emergency dispatches in Okayama, Japan, from January 2017 to December 2022.

## Material and Methods

### Participants and procedure

Using data from emergency dispatches, we included individuals who attempted or completed suicide in Okayama City and Kibichuo Town in Okayama Prefecture from January 2017 to December 2022.

We first obtained electronic data for all ambulance calls from January 2017 to December 2022 from the Emergency Section of the Fire Bureau in Okayama City. We restricted our analyses to emergencies that occurred in Okayama City and Kibichuo Town in Okayama Prefecture (N = 198,361). Furthermore, we restricted the data to emergencies that required the dispatch of an ambulance to the scene, leaving 197,121 emergencies. The electronic data included the time of the emergency call, location of occurrence, sex and age of the involved person, and incident type. The incident type refers to the reason for the emergency call and includes acute disease, general injury, traffic accident, fire, suicide attempt, and homicide. Emergency medical technicians recorded and corrected all information, as necessary.

Subsequently, we focused on suicide attempts in which the incident type was recorded. We included all types of suicide attempt, which comprised not only individuals who died from suicide but also those who survived a suicide attempt. We excluded all other emergencies except those involving suicide attempts, leaving 1,801 emergencies.

### COVID-19 pandemic period

In the present study, we defined the COVID-19 pandemic as the period from January 2020 to December 2022; we used this period as the first patient in Japan was identified in January 2020, and several restrictions imposed on the Japanese population started at that time. In addition, we used the 3 years prior to the pandemic (i.e., January 2017 to December 2019) as the control period.

### Statistical analysis

First, descriptive analysis was conducted, and the numbers of suicide attempts, as well as other variables, were compared between the periods before and during the COVID-19 pandemic. To evaluate changes in the suicide attempt rates between the periods before and during the pandemic, interrupted time-series analysis was conducted using generalized Poisson regression models. The daily number of suicide attempts was counted for the total population as well as by sex and age group (15-24, 25-49, 50-64, and ≥65 years). The corresponding population size, obtained from the 2020 census, was included as an offset variable ^(14)^. The daily number of suicide attempts was regressed on the indicator of the pandemic period, and the rate ratios (RRs) with their 95% confidence intervals (CIs) were estimated for suicide attempts during the pandemic period (2020-2022) compared with the control period (2017-2019). After estimating the crude RRs, the day of the week (categorical), public holidays (dichotomous), daily average temperature (continuous) ^(15)^, and calendar month (categorical) were adjusted. The daily average temperature was included as a covariate owing to its reported association with suicide ^(16)^. A restricted cubic spline with five knots was used for temperature in the model. The daily average temperatures for Okayama City were obtained from the Japan Meteorological Agency.

In an additional analysis, the pandemic period was divided into 1-year intervals, and the rates of suicide attempt in each year were compared with those in the control period using the same model.

Stata SE Version 18.1 (StataCorp LLC, College Station, TX, USA) was used for all analyses. The study was approved by the Institutional Review Board of Okayama University Graduate School of Medicine, Dentistry, and Pharmaceutical Sciences (2003-029). The requirement for informed consent was waived due to the retrospective nature of the study, and participant anonymity was assured.

## Results

Descriptive statistics are presented in Table 1. The number of dispatches for suicide attempts increased during the pandemic period compared with the control period. Similar patterns were found for the number of suicide attempts according to sex and in some age categories.

**Table 1.** Descriptive Statistics for the Pre-COVID-19 Pandemic Period (Control Period) and the Pandemic Period.

	Control period (From Jan 2017 to Dec 2019)	COVID-19 pandemic period (From Jan 2020 to Dec 2022)
Number of suicide attempts
Total	843	958
Men	298	353
15-24	46	51
25-49	125	160
50-64	71	62
≧65	50	75
Women	527	595
15-24	129	145
25-49	239	297
50-64	83	86
≧65	67	61
Number (%) of suicides completed within 1 month after attempts	91 (10.8)^a^	85 (8.9)^a^
Temperature, mean (SD)	16.2 (8.7)	16.5 (8.5)
Holidays, n (% in each period)	59 (5.4)	51 (4.7)

SD, standard deviation Information on sex was unknown for 18 individuals in 2017-2019 and for 10 individuals in 2020-2022. Information on age was unknown for 15 individuals in 2017-2019 and for 10 individuals in 2020-2022. There were 11 and 9 individuals aged less than 15 years in 2017-2019 and 2020-2022, respectively. ^a^ Proportions among those who attempted suicide

Compared with the control period, the rates of suicide attempt increased during the pandemic period. The RR for suicide attempts was 1.13 (95% CI: 1.03-1.24) in total, 1.17 (95% CI: 1.00-1.37) in men, and 1.12 (95% CI: 1.00-1.26) in women. In particular, these rates increased among men aged 25-49 years and ≥65 years as well as among women aged 25-49 years (Table 2).

**Table 2.** Rate Ratios for Suicide Attempts during the COVID-19 Pandemic Compared with the Prepandemic Period (Control Period).

	Crude RRs (95% CI)^a^	Adjusted RRs (95% CI)^a^
Both men and women	1.13 (1.03-1.24)	1.13 (1.03-1.24)
Men	1.18 (1.01-1.38)	1.17 (1.00-1.37)
15-24	1.11 (0.74-1.65)	1.11 (0.75-1.66)
25-49	1.28 (1.01-1.62)	1.27 (1.00-1.60)
50-64	0.87 (0.62-1.23)	0.85 (0.60-1.19)
≧65	1.50 (1.05-2.14)	1.53 (1.07-2.18)
Women	1.13 (1.00-1.27)	1.12 (1.00-1.26)
15-24	1.12 (0.89-1.42)	1.12 (0.89-1.43)
25-49	1.24 (1.05-1.47)	1.23 (1.04-1.46)
50-64	1.04 (0.77-1.40)	1.02 (0.75-1.38)
≧65	0.91 (0.64-1.29)	0.91 (0.64-1.29)

^a^ The RRs were estimated in comparison with the pre-COVID-19 period (Jan 2017-Dec 2019). The RRs were estimated, adjusting for day of the week, holidays, month, and mean temperature. CI, confidence interval; RR, rate ratio

**Figure 1.**
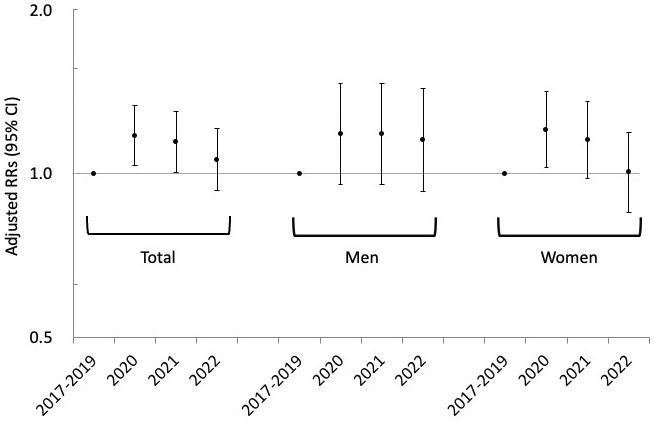
Rate ratios for suicide attempts in each year (2020, 2021, and 2022) during the COVID-19 pandemic compared with the prepandemic period (control period, 2017–2019). The RRs were estimated in comparison with the pre-COVID-19 period (January 2017–December 2019). The RRs were estimated, adjusting for day of the week, holidays, month, and mean temperature. CI, confidence interval; RR, rate ratio.

When the pandemic period was divided into 1-year intervals, the risk increased only for the years 2020 and 2021 compared with the prepandemic period. No increased risk was observed in 2022 (Figure 1).

## Discussion

In this study, we observed an increase in the number of suicide attempts during the COVID-19 pandemic compared with the control period. The observed increase in the rates of suicide attempt in 2020 is consistent with the findings of previous studies indicating increases in suicide mortality and attempts during 2020 ^(5), (7), (8)^. Furthermore, the present study supports previous findings of increased suicide mortality during 2021, with our results indicating that suicide attempts also increased during 2021 ^(9), (10)^.

The suicide attempt rates increased among men aged 25-49 and ≥65 years, which is partly consistent with the findings of previous studies ^(7), (10)^. The reason for these increased rates during the pandemic could be associated with work-related stress or economic pressures in the age group 25-49 years as well as decreased opportunities to meet and communicate with others among older people (age ≥65 years) ^(11), (17), (18)^. In addition, the suicide attempt rates increased among women aged 25-49 years, which is consistent with the findings of previous studies ^(7), (8), (9), (10)^. Several published studies have reported that the reasons for such increases in these age groups are associated with stress related to an increased burden of childcare, employment insecurity, and decreased income ^(11), (19)^.

The strength of the present study is that we analyzed individual data rather than aggregated data, as used in previous studies ^(7), (9), (10), (11)^. This allowed us to adjust for important confounders in the analysis. Moreover, there were no environmental changes that affected the flow of patient referrals in the target or surrounding areas during the study period. This study also has several limitations that need to be acknowledged. First, we could not include cases of suicide that did not require an ambulance call, which may have underestimated the number of suicides and suicide attempts. Second, we only included participants from specific areas of Okayama, which may limit the generalizability of our findings. Third, we did not obtain information on the background or specific reasons for each suicide attempt.

In conclusion, we found that the rate of suicide attempts increased during the COVID-19 pandemic, particularly in 2020 and 2021, compared with the prepandemic period. Such an increase was more pronounced among men aged 25-49 years and ≥65 years and among women aged 25-49 years. Greater attention should be given to these vulnerable groups to prevent suicide attempts during a public health emergency, such as the COVID-19 pandemic. Future studies are warranted, expanding the study area to all of Japan and focusing on the long-term impacts of the COVID-19 pandemic on suicide attempts, particularly among vulnerable groups.

## Article Information

### Conflicts of Interest

None

### Sources of Funding

This work was supported by [JSPS KAKENHI] grant number [20K10499].

### Acknowledgement

We thank Saori Irie and Yoko Oka for their valuable support in collecting the data and writing the manuscript. We thank Analisa Avila, MPH, ELS, of Edanz (https://jp.edanz.com/ac) for editing the draft of this manuscript.

### Author Contributions

Ms. Yamamura conceptualized and designed the study, conducted the initial analyses, drafted the initial manuscript, and reviewed and revised the manuscript.

Drs. Matsumoto and Takao conceptualized and designed the study and reviewed and revised the manuscript.

Prof. Yorifuji conceptualized and designed the study and critically reviewed the manuscript for important intellectual content.

All authors approved the final submitted manuscript and agree to be accountable for all aspects of the work.

### Approval by Institutional Review Board (IRB)

The study was approved by the Institutional Review Board of Okayama University Graduate School of Medicine, Dentistry, and Pharmaceutical Sciences (2003-029).
